# A nearly telomere-to-telomere diploid genome assembly of *Firmiana kwangsiensis*, a threatened species in China

**DOI:** 10.1038/s41597-024-04250-8

**Published:** 2024-12-18

**Authors:** Boqiang Wang, Rengang Zhang, Weibang Sun, Jing Yang

**Affiliations:** 1 Yunnan Key Laboratory for Integrative Conservation of Plant Species with Extremely Small Populations, Kunming Institute of Botany, Chinese Academy of Sciences, Kunming, 650201 China; 2 University of Chinese Academy of Sciences, Beijing, 101408 China; 3 Key Laboratory for Plant Diversity and Biogeography of East Asia, Kunming Institute of Botany, Chinese Academy of Sciences, Kunming, 650201 China; 4 Kunming Botanical Garden, Kunming Institute of Botany, Chinese Academy of Sciences, Kunming, 650201 Yunnan China

**Keywords:** Conservation genomics, Plant genetics, Plant genetics

## Abstract

*Firmiana kwangsiensis* is a tree species of high ornamental value. The species is critically endangered in the wild, and is listed as a first-class national protected wild plant in China, and a Plant Species with Extremely Small Populations in need of urgent protection. We have assembled a chromosome-scale, haplotype-resolved genome for *F. kwangsiensis* using a combination of PacBio HiFi sequencing, ONT sequencing, and Hi-C sequencing. The final assembled genome is 2.3 G in size and comprises 2n = 40 chromosomes. All chromosomal ends contain telomeric characteristic motifs (TTTAGGG), and there are only 2 gaps within the rDNA regions, both close to a T2T genome assembly. Two complete sets of haplotypes are present, Haplotype A (1169.19 Mb) and Haplotype B (1157.87 Mb), with contig N50 lengths of 58.37 Mb and 57.27 Mb, respectively. The genome contains a total of 67,527 coding genes, with 62,351 genes functionally annotated here. This is the first report of the genome of *F. kwangsiensis*, and lays the foundation for future conservation genomics research into this species.

## Background & Summary

The woody genus *Firmiana* Marsili (Malvaceae) includes 18 species, which are distributed discontinuously in the tropical to subtropical regions of eastern and southeastern Asia, as well as eastern Africa^1,2^. Ten of these species have a natural distribution that includes the tropical and subtropical areas of China, and seven are endemic to this region. Most of the fossil records of *Firmiana* have been discovered in East Asia. A specimen has recently been found from the middle Eocene deposits of central Tibet, demonstrating that this genus also occurred in that area^3^. *Firmiana* is thought to have radiated tens of millions of years ago^4–6^ and was likely to have been very abundant at this time. However, the genus is now endangered throughout its modern distribution. In 2021, all Chinese members of the genus (with the exception of *F. simplex*) were listed as National Protected Wild Plants^7^.

**Fig. 1:**
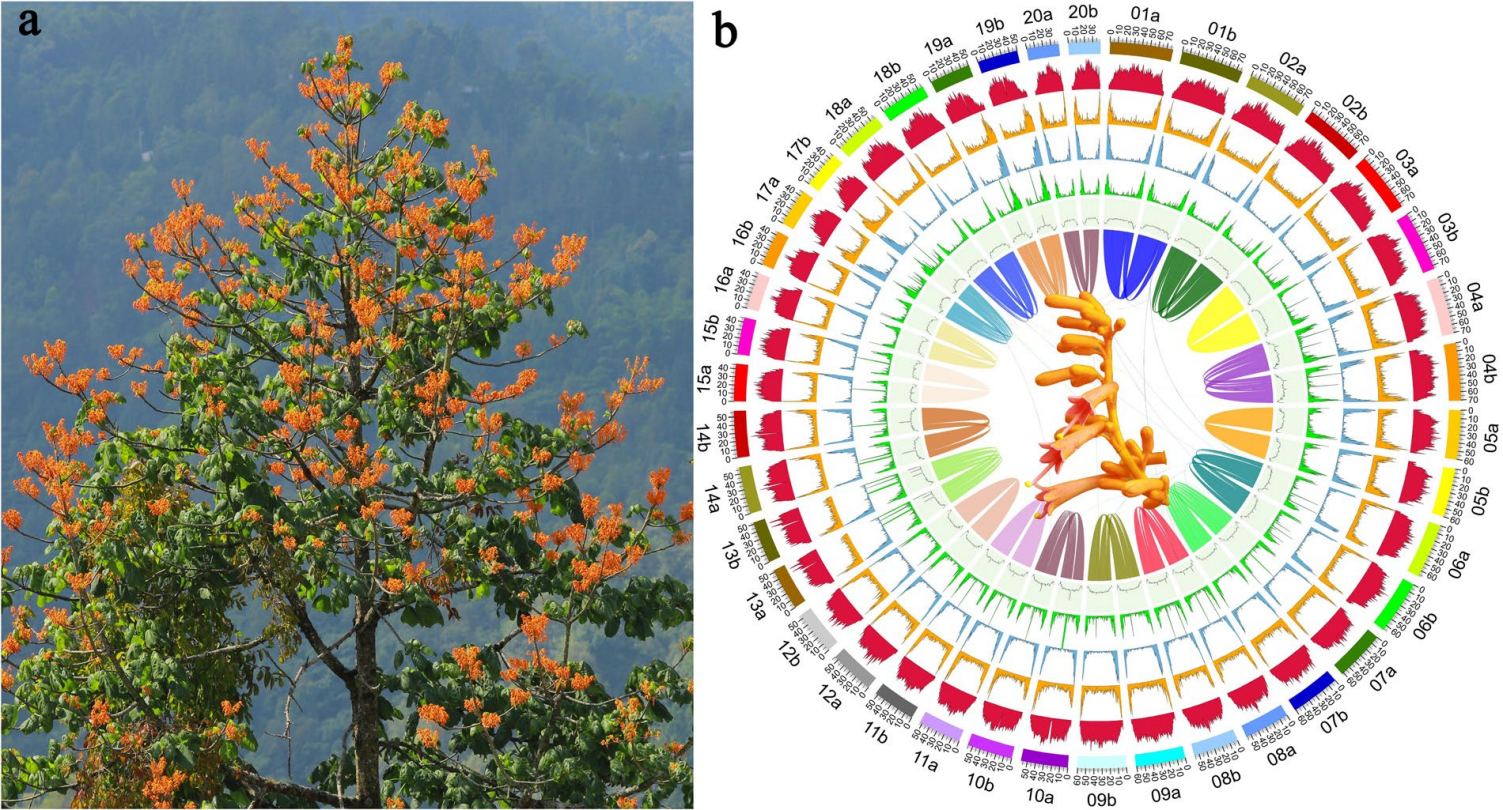
(**a**) Illustration of the morphology of *Firmiana kwangsiensis*; (**b**) Presentation of a genomic overview, including from outer to inner circles: pseudo-chromosomes, density of retrotransposons (Class I TEs), density of DNA transposons (Class II TEs), protein-coding gene density, proportion of tandem repeats, GC content, and collinearity blocks.

*Firmiana kwangsiensis*, which is endemic to the limestone regions of Guangxi in southern China, stands out on account of its striking orange-red blossoms and distinctive palmate leaves, potentially making it a valuable ornamental tree species (Fig. 1a,b). However, despite its aesthetic appeal, this species faces extinction, primarily due to its limited natural regeneration capabilities and the ongoing loss of its natural habitat^8^. Recognized by the International Union for Conservation of Nature (IUCN) as critically endangered (CR), *F. kwangsiensis* is also featured on China’s Biodiversity Red List and was designated a Chinese national key protected wild plant in 2021^9^. In a proactive move, the Chinese State Forestry and Grassland Administration has prioritized *F. kwangsiensis* within the “14th Five-Year Plan” National Program for the Rescue and Protection of plant species with extremely small populations (PSESP). These species are marked by a restricted geographical range, susceptibility to prolonged external disturbances, and a critically low number of individuals^10^. With a wild population not exceeding 5,000 mature individuals and a maximum of 500 mature individuals per isolated population9, the urgency of conservation is clear.

The *de novo* assembly of plant genomes is pivotal for advancing our comprehension of plant evolutionary processes, the breeding of ornamental species and conservation efforts^11–14^. Here, we assembled a nearly telomere-to-telomere genome of *F. kwangsiensis* using PacBio HiFi sequencing, whole genome ONT sequencing, and Hi-C sequencing. The final assembled genome was found to be 2.3 G in size with only 2 gaps within rDNA regions, indicating good completeness, and was close to a telomere-to-telomere gapless genome. Two complete sets of haplotypes were found (Fig. 1b), Haplotype A (1169.19 Mb) and Haplotype B (1157.87 Mb), with contig N50 lengths of 58.37 Mb and 57.27 Mb, respectively (Table 1). All the telomeric ends of the chromosomes have assembled with the telomeric signature sequence (TTTAGGG)_n_. The mitochondrial genome was 756,902 bp in size, and the chloroplast genome was 160,834 bp in size. A total of 1,989,057 repeat sequences were identified, with a total length of 1,749,627,480 bp, accounting for 75.19% of the genome. Of these, the most abundant were found to be long terminal repeat retrotransposons (LTR-RTs), with a total of 1,233,393 elements, accumulating a length of 1,613,986,623 bp, and constituting 69.36% of the total repetitive sequences (Table 2). The genome of the *F. kwangsiensis* contains a total of 79,839 genes, comprising 67,527 protein-coding genes and 12,312 non-coding genes (Table 3). A total of 62,351 protein-coding genes were functionally annotated (Table 4). This study obtained a high-quality genome for *F. kwangsiensis*, which will be of great significance in the conservation of this species.

**Table 1:** Summary of *F. kwangsiensis* genome assembly.

Parameter	Genome	Haplotype A	Haplotype B
Genome size	2,327,055,126 bp	1,169,189,033 bp	1,157,866,093 bp
GC content	37.43%	37.45%	37.41%
Contig number	45	24	21
Contig N10	74,570,186 bp	74,680,766 bp	74,395,429 bp
Contig N50	57,867,992 bp	58,369,891 bp	57,274,153 bp
Contig N90	46,251,825 bp	46,251,825 bp	45,832,173 bp
Scafold number	43	23	20
Scafold N10	74,570,186 bp	74,680,766 bp	74,395,429 bp
Scafold N50	57,867,992 bp	58,369,891 bp	57,274,153 bp
Scafold N90	46,251,825 bp	46,251,825 bp	45,832,173 bp
Gap number	2	1	1

**Table 2:** Summary of repeat elements.

Type	Number	Length (bp)	Percent (%)	Mean length (bp)
LTRs	1,233,393	1,613,986,623	69.36	1,308.57
LINE	3,812	2,010,257	0.09	527.35
Helitron	35,240	10,412,187	0.45	295.47
TIR	129,328	67,554,276	2.9	522.35
Unknown	104,454	33,354,807	1.43	319.33
Simple repeats	396,466	17,728,780	0.76	44.72
Low complexity	86,364	4,580,550	0.2	53.04
Total	1,989,057	1,749,627,480	75.19	879.63

**Table 3:** Summary of *F. kwangsiensis* genome annotations.

Feature		Total	Haplotype A	Haplotype B
gene		79,839	40,259	39,580
transcript		102,987	51,910	51,077
exon		550,260	276,602	273,658
intron		447,273	224,692	222,581
exons/transcript		102,987	51,910	51,077
mRNA	gene	67,527	33,883	33,644
	transcript	90,675	45,534	45,141
tRNA		1,064	553	511
rRNA		750	487	263
ncRNA		10,498	5,336	5,162

**Table 4:** Functional annotation of protein-coding genes in *F. kwangsiensis*.

Program	Database	Gene number	Percentage (%)
eggNOG-mapper	GO	30,308	44.88
	KEEG_Pathway	17,591	26.05
	KEEG_KO	28,215	41.78
	eggNOG	55,608	82.35
	COG	60,261	89.24
	EC	12,214	18.09
DIAMOND	Swiss_Prot	45,520	67.41
	TrEMBL	61,236	90.68
	NR	60,868	90.14
	*A*. *thaliana*	54,826	81.19
InterProScan	Pfam	50,075	74.16
	CDD	21,360	31.63
	PRINTS	8,724	12.92
	Interpro	52,398	77.6
	Phobius	22,355	33.11
	Gene3D	42,127	62.39
	SUPERFAMILY	39,303	58.2
	TIGRFAM	6,162	9.13
	Coils	10,525	15.59
	SMART	19,326	28.62
	Unannotated	5,176	7.67

## Methods

### Plant material

For the purpose of sequencing, fresh leaf samples were carefully collected from a robust *Firmiana kwangsiensis* individual, which was growing *ex situ* in Kunming Botanical Garden of the Kunming Institute of Botany (Chinese Academy of Sciences). This particular plant was originally procured from Guilin, Guangxi, and was transplanted to the garden in September 2016. In addition to leaves collected, stems, roots, and buds were also harvested to facilitate comprehensive transcriptome sequencing. Samples were quickly immersed in liquid nitrogen post-collection, to arrest cellular processes and preserve the integrity of the nucleic acids. Samples were subsequently stored in dry ice until further processing. Sequencing was carried out by a commercial sequencing service provider, Wuhan Benagen Technology Co. Ltd., of Wuhan, China.

### PacBio HiFi sequencing

High-quality genomic DNA was extracted from the fresh leaves of *F. kwangsiensis* using a CTAB method^15^, and DNA quality and concentration were tested using 0.75% agarose gel electrophoresis, a NanoDrop One spectrophotometer (Thermo Fisher Scientific, USA), and a Qubit 3.0 Fluorometer (Life Technologies, USA). The libraries used for single-molecule real-time (SMRT) Pacific Biosciences (PacBio) genome sequencing were constructed according to standard protocols. A library with a DNA-fragment insert size of ~15 kb was prepared from 3 μg of high-quality genomic DNA. Before uploading, the libraries were prepared using the PacBio Binding kit (Pacbio USA), and were purified with AMpure PB Beads (Pacbio, USA), and finally placed into the Revio system (Pacbio, USA) sequencer for sequencing. A total of 84 Gb (~ 4.5 M reads) of HiFi data were produced, implying ~73 × genome coverage. The average read length was 19 kb, and the N50 was 19 kb (Supplementary Table 1).

### ONT (Oxford Nanopore Technologies) sequencing

After the DNA quality and integrity was tested, the inserts were randomly sheared using a Covaris ultrasonic disruptor. Libraries were prepared using the SQK-LSK109 ligation kit, according to standard protocols. Purified libraries were loaded onto primed R9.4 Spot-On Flow Cells and were sequenced using a PromethION sequencer (Oxford Nanopore Technologies, UK). Raw data were analyzed for base calling using the Oxford Nanopore GUPPY software (v0.3.0). This produced 47 Gb (~ 1.6 M reads) of ONT data, which implies ~41 × genome coverage. The average read length was 29 kb, and the N50 was 65 kb (Supplementary Table 2).

### Hi-C sequencing

Plant tissues were crosslinked in a vacuum with 2% formaldehyde solution at room temperature, according to the standard protocol of Benetech Ltd^16^, and Gly was then added to quench the crosslinking reaction. The cross-linked chromatin was enzymatically digested using 400 units of MboI, and labelled with biotin-14-dCTP. Subsequently, blunt-end ligation of the cross-linked DNA fragments was conducted using a ligation enzyme. After purification of the DNA and removal of biotin-c from the ends of non-ligated fragments, the fragments were sheared to a size of 200–600 basepairs with sonication and the fragment ends were repaired. The Hi-C libraries were enriched, A-tailed, and then subjected to PCR amplification (12–14 cycles). Subsequently, the Hi-C libraries were sequenced using the DNBSEQ-T7 platform (BGI Inc., China), operating in PE150 mode. Approximately 125 Gb (~832 M reads) of Hi-C data were generated for subsequent pseudochromosome assembly (Supplementary Table 3).

### Full-length transcriptome sequencing

Total RNA was extracted using the R6827 Plant RNA Kit (Omega Bio-Tek, Norcross, GA, USA) and RNA samples were examined using a NanoDrop One spectrophotometer (NanoDrop Technologies, DE) and a Qubit 3.0 Fluorometer (Life Technologies, Carlsbad, CA, USA). Libraries were then prepared using a PCR-cDNA sequencing kit (SQK-PCS109) and a PCR Barcoding kit (SQKPBK004), and were sequenced on a PromethION sequencer^17^ (Oxford Nanopore Technologies, UK). Finally, a total of 19 Gb (~16 M reads) of full-length RNA-seq data was obtained (Supplementary Table 4).

### Estimation of genome size and heterozygosity analysis

The 19-mer frequency distribution was calculated using KMC (v3.2.4)^18^, and we finally estimated genome size, heterozygosity, and degree of duplication based on the kmer frequency distribution using GCE (v. 1.0.0)^19^. Moreover, we also combined findGSE (v0.1.0)^20^ and genomescope (v2.0)^21^ for evaluation. Taken together, the genome size of *F. kwangsiensis* was estimated to be about 1.2 Gb (1 C size), with a heterozygosity of about 0.8% (Supplementary Table 5 and Supplementary Figure 1).

### Genome assembly

Using a recent version of the assembler Hifiasm (v0.19.8-r602)^22^, HiFi reads, ONT ultra-long reads and Hi-C reads were combined to generate a haplotype-resolved assembly. Juicer (v1.5.6)^23^ was then used to map the Hi-C reads to this assembly and 3D-DNA (v180922)^24^ (with the parameters–early-exit -m haploid -r 0) was used to scaffold chromosome-level genome, which was manually tuned with Juicebox (v1.11.08)^25^ (pre -n -q 0 or 1) to correct and adjust the mis-assemblies, chromosome boundaries and switch errors. The 3D-DNA scaffolding pipeline was used again on each chromosome separately. Final chromosome scaffolds and un-anchored sequences were obtained after manual refinements in Juicebox, through removal of errors such as wrong placements and orientations.

Together with the HiFi reads, the GapFiller module in the quarTeT (v1.1.2)^26^ was utilized to fill unclosed gaps. The characteristic telomere motif (TTTAGGG) was found toward both ends of most chromosomes. For those that were short or lacked this region, HiFi reads were aligned back to their target chromosomes and the ends were assembled to contigs using Hifiasm (v0.19.8-r602). By mapping the contigs back to chromosomes, the extended parts were assigned in the assembly of telomeres. The chloroplast and mitochondrial genomes were assembled using the GetOrganelle toolkit (v1.7.5)^27^. HiFi reads was mapped to the unitig graph of the mitochondrial assembly using minimap2 (v2.24)^28^ to resolve repeat nodes, with manual adjustment using Bandage (v0.9.0)^29^. Redundans (v0.13c)^30^ (with the parameters -identity 0.98 -overlap 0.8) was used to map un-anchored contigs to the chromosome and organelle genome sequences, and redundancies such as low-coverage fragments, haplotigs and rDNA fragments in un-anchored sequences were identified and removed with manual curation.

### Comparison of haplotype assemblies

**Fig. 2:**
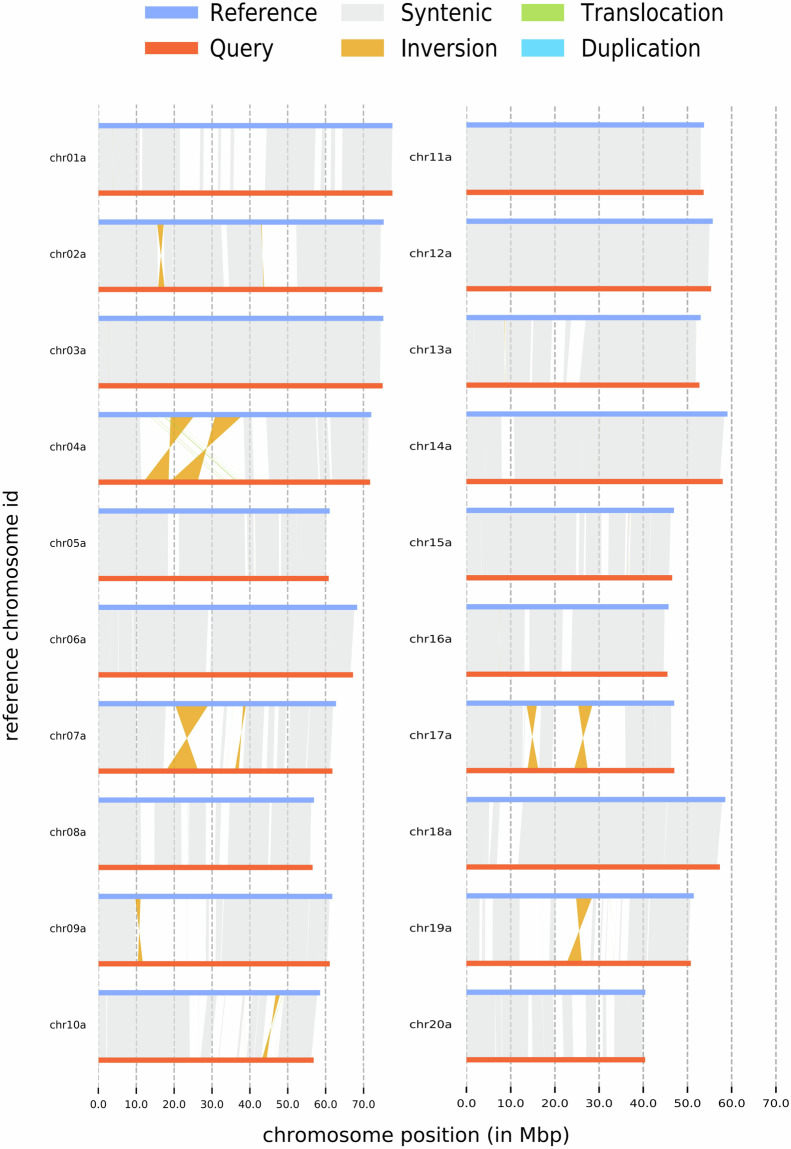
Depiction of the genome architecture contrast between haplotypes A and B, with haplotype A serving as the reference and haplotype B being utilized as the query.

Employing SyRI (v1.646) with the default parameters, we conducted a detailed analysis to uncover the synteny and structural variations between the two identified haplotypes. The analysis identified 177 syntenic regions (~938 Mb), 49 inversions (38 Mb) and 137 translocations (~1 Mb). Notably, Haplotype A exhibited 32 duplications (~0.2 Mb), while Haplotype B had a higher incidence, with 119 duplications (~0.7 Mb). The majority of inversions were detected on chromosomes 2, 4, 7, 9, 10, 17, and 19, with translocations predominantly occurring on chromosome 4 (Fig. 2). Additionally, we detected 2,320,698 SNPs (~2 Mb), 155,633 insertions (~13 Mb in Haplotype B), 154,536 deletions (~12 Mb in Haplotype A), and 12 tandem repeats (~0.005 Mb). These findings provide valuable insights into the genetic diversity and structural plasticity of the haplotypes under study.

### Identifcation of repetitive elements

*De novo* identification of transposable elements (TEs) was performed using EDTA^31^ (v. 1.9.9; parameters:–sensitive 1–anno 1) and TE libraries were generated. Repeat elements in the assembled genome were then identified with RepeatMasker (v4.0.7) (with the parameters -no_is -xsmall) (http://www.repeatmasker.org/RepeatMasker/). We identified a total of 1,989,057 repetitive sequences with a total length of 1,749,627,480 bp, accounting for 75.19% of the genome. The highest proportion of the repetative sequences were found to be LTR, accounting for 69.36% of the genome Table 2).

### Genome annotation

The RNA-seq reads were mapped to the genome using minimap2 (v2.24)^28^ (with the parameters -a -x splice –end-seed-pen = 60 –G 200k), and were then assembled using StringTie (v2.1.5)^32^ (with the parameters -L -t -f 0.05). The publicly available combined 358,383 non-redundant protein sequences (including *Bombax ceiba*^33^, *Theobroma cacao*^34^, *Durio zibethinus*^35^, *Corchorus capsularis*^36^, *Gossypium raimondii*^37^, *Heritiera littoralis*^38^, *Ochroma pyramidale*^39^, *Firmiana major*^40^, *Dipterocarpus turbinatus*^41^, *Aquilaria sinensis*^42^, *Arabidopsis thaliana*^43^, *Carica papaya*^44^, and *Vitis vinifera*^45^) were used for evidence of homologous proteins in our gene annotation. Based on the transcript evidence, the gene structure was annotated using the PASA (v2.4.1) process^46^, and then the full-length genes were identified by mapping them to reference proteins. Finally, based on the full-length gene set, AUGUSTUS (v3.4.0)^47^ was trained and optimized in 5 rounds.

We used MAKER2 (v2.31.9)^48^ to annotate based on ab initio predictions, transcript evidence, and homologous protein evidence. First, repetitive sequences were masked using RepeatMasker, and AUGUSTUS and SNAP^49^ were used for ab initio coding gene prediction. Then the transcript sequences and protein sequences were aligned to the repeat-masking genome using BLASTN and BLASTX, respectively, and the alignment was optimized using Exonerate (v2.2.0)^50^. Finally, based on the above evidence, the hints files were generated and then AUGUSTUS and SNAP was integrated to predict the gene models.

As MAKER process annotations have low precision^51^, we further integrated MAKER and PASA annotations with EVidenceModeler (v1.1.1)^52^ (EVM) to generate consistent gene annotations. Furthermore, to avoid introducing TE coding regions, TE protein domains on the genome were identified with TEsorter (v1.4.15)^53^ and masked with EVM. The EVM annotations were then upgraded with PASA to add UTRs and alternative splicing transcripts. Genes with abnormal coding frames and excessively short (<50 aa) genes were removed. While annotating non-coding RNA, we annotated tRNA with tRNAScan-SE (v1.3.1)^54^ and rRNA with Barrnap (v0.9) (https://github.com/tseemann/barrnap). RfamScan (v14.2)^55^ was used for comparative annotation of various noncoding ncRNAs. In the end, all the annotation results were merged and redundancies were removed to get the complete set of genes.

A total of 67,572 protein-coding genes were predicted in our analyses, with an average length of 3,892 bp, and comprising 90,675 transcripts and 537,896 exons (Table 3). Furthermore, 10,498 non-coding genes were identified, including 750 rRNAs, 1,064 tRNAs and other ncRNAs.

We used three strategies to predict gene function. We used eggNOG-mapper (v2.0.0)^56^ (–target_taxa Viridiplantae -m diamond) to compare with the database of eggNOG homologous genes (including GO, KEGG, and others) to annotate the function of the genes. DIAMOND (v2.0.4)^57^ (Identity > 30%, E-value < 1e-5) was used to align protein sequences to protein databases (e.g. Swiss_Prot, TrEMBL, NR and the Arabidopsis database), to identify the best hits of genes. We also used InterProScan (v5.27-66.0)^58^ to match the sub-databases PRINTS, Pfam, SMART, PANTHER, CDD, and others, to obtain the conserved motifs and domains of the proteins (Table 4).

## Data Records

The raw sequencing data have been deposited in the Sequence Read Archive database of NCBI (National Center for Biotechnology Information) under accession numbers SRR30515156-SRR30515159^59–62^. The genome assembly files and the genome annotation files have been submitted to Figshare^63^. The genome assembly data can also be accessed at GenBank using the accession number JBHMQQ000000000-JBHMQR000000000^64,65^.

## Technical Validation

### Evaluation of the assembled genome

**Fig. 3:**
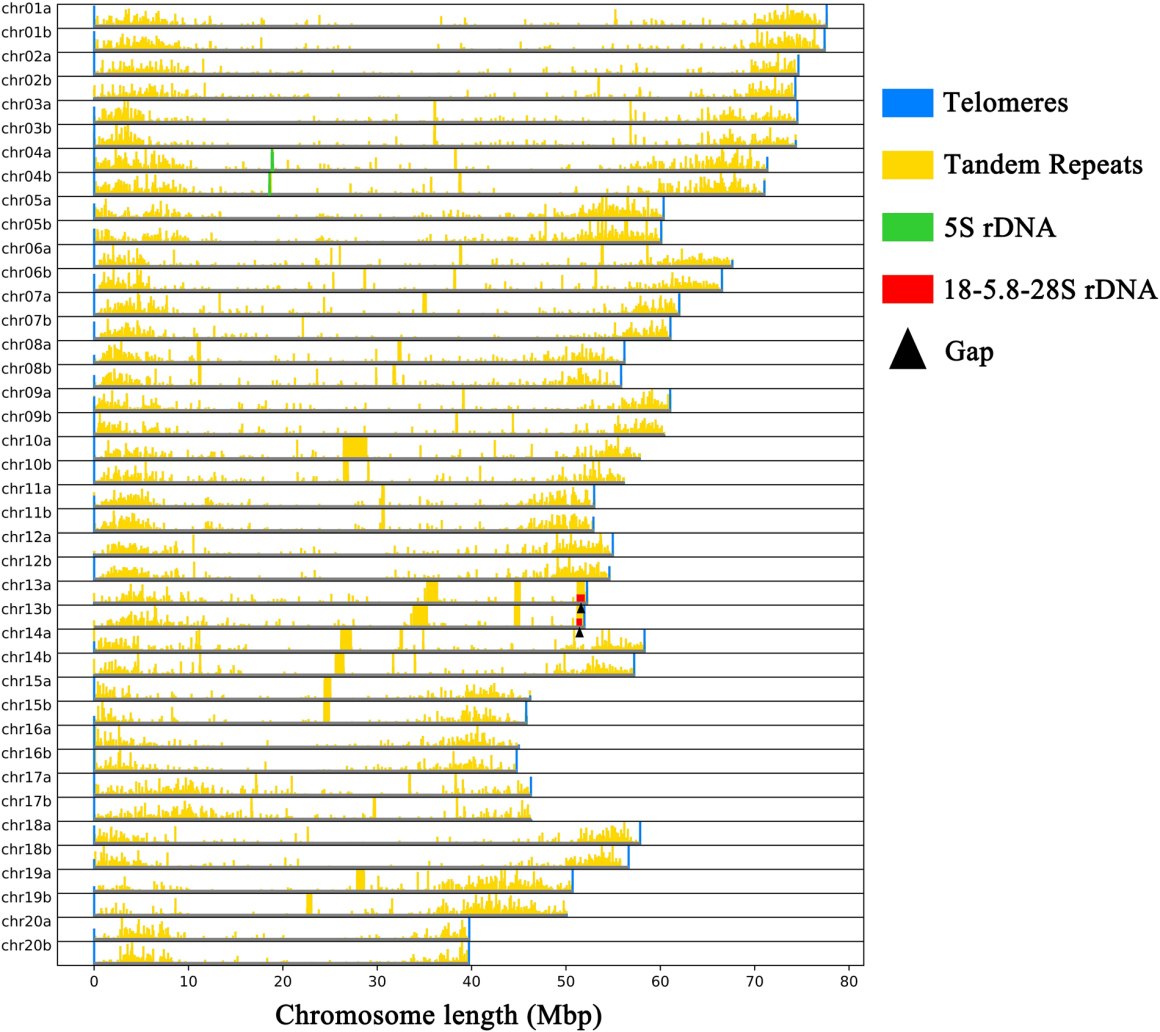
Profiles of repetitive sequence distribution across the chromosomes of *Firmiana kwangsiensis*, highlighting telomeric TTTAGGG sequences, tandem repeats, 5S rDNA, and 45S rDNA. The vertical scale denotes the frequency of repetitive sequences in 20 kb segments. Black triangles signify the positions of gaps.

**Fig. 4:**
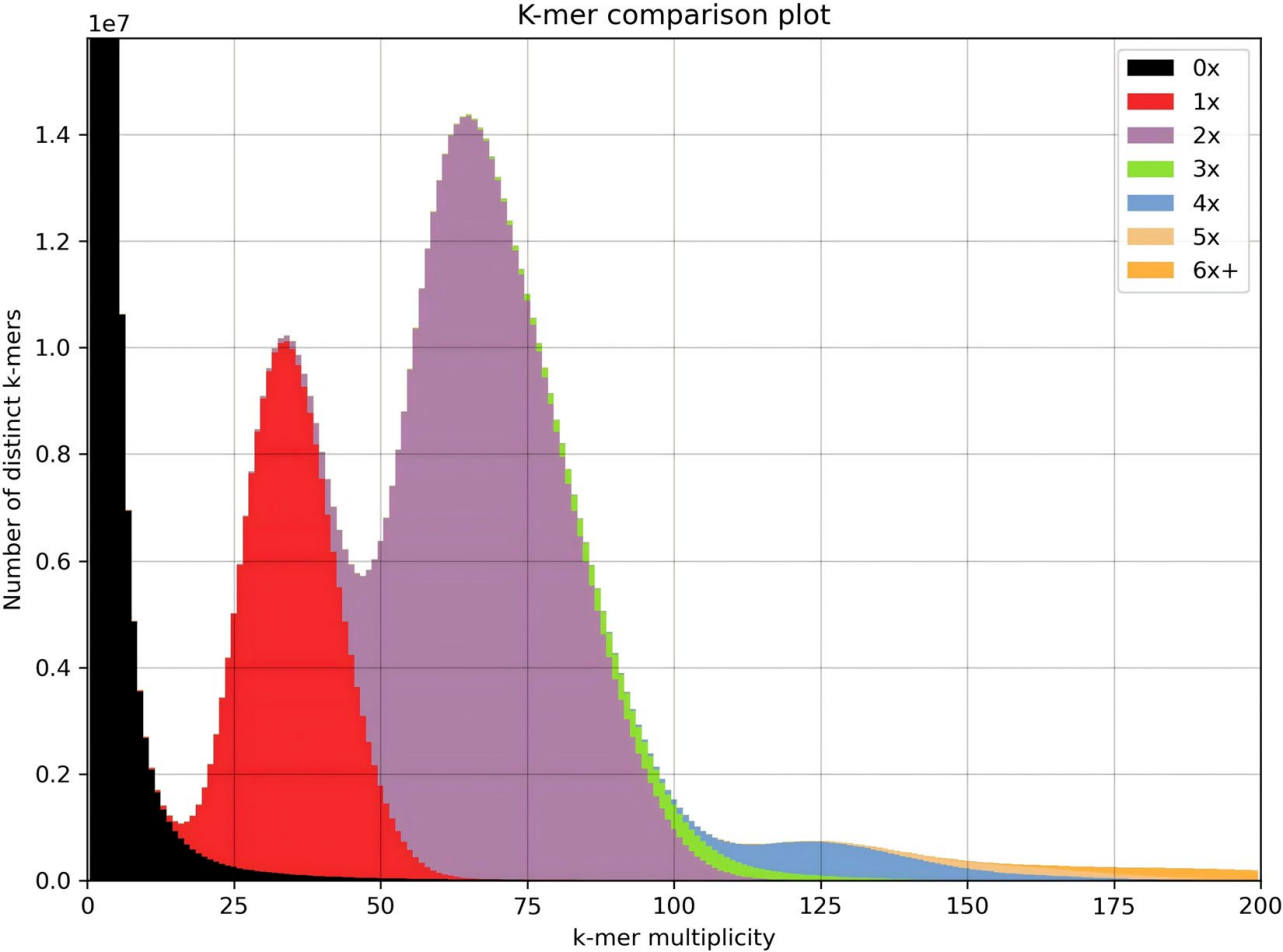
Graphical representation of copy number variation spectra for genome assembly validation against HiFi sequencing data.

In the genome assembly of *F. kwangsiensis*, we found two complete haplotypes, with a total size of 2.3 Gb. Telomere sequences (TTTAGGG) were identified in all the chromosomal ends, while 45S and 5S rDNA arrays were detected on Chr13 and Chr4, respectively (Fig. 3). Each haplotype has only one gap, located on chromosome 13 on the 45S rDNA regions (Fig. 3). Despite significant advancements in genome sequencing and assembly techniques, the intricacy of rDNA regions continues to pose challenges for complete resolution and assembly^66^. Therefore, the fact that our assembled genome exhibits only two gaps within the 45S rDNA regions, suggests that the assembly quality is nearly on par with that of a telomere-to-telomere (T2T) genome. The HiFi reads and the RNA sequencing reads were mapped to the assembly using Minimap2 (v2.24) and HISAT2 (v2.1.0) respectively. The quality was also validated by the high mapping rates achieved (Supplementary Table 6), with the results showing that 99.92% of the assembly was covered at least 10-fold by the HiFi reads. By plotting the distribution of coverage depth across the whole genome against the HiFi data (Fig. 4), we found that that the assembly had neither redundancy nor homologous collapse. Furthermore, the quality of the final assembly was assessed using BUSCO^67^ (v5.3.2), which resulted in a high completeness score of 98.8% for *F. kwangsiensis* (Supplementary Table 7).

**Fig. 5:**
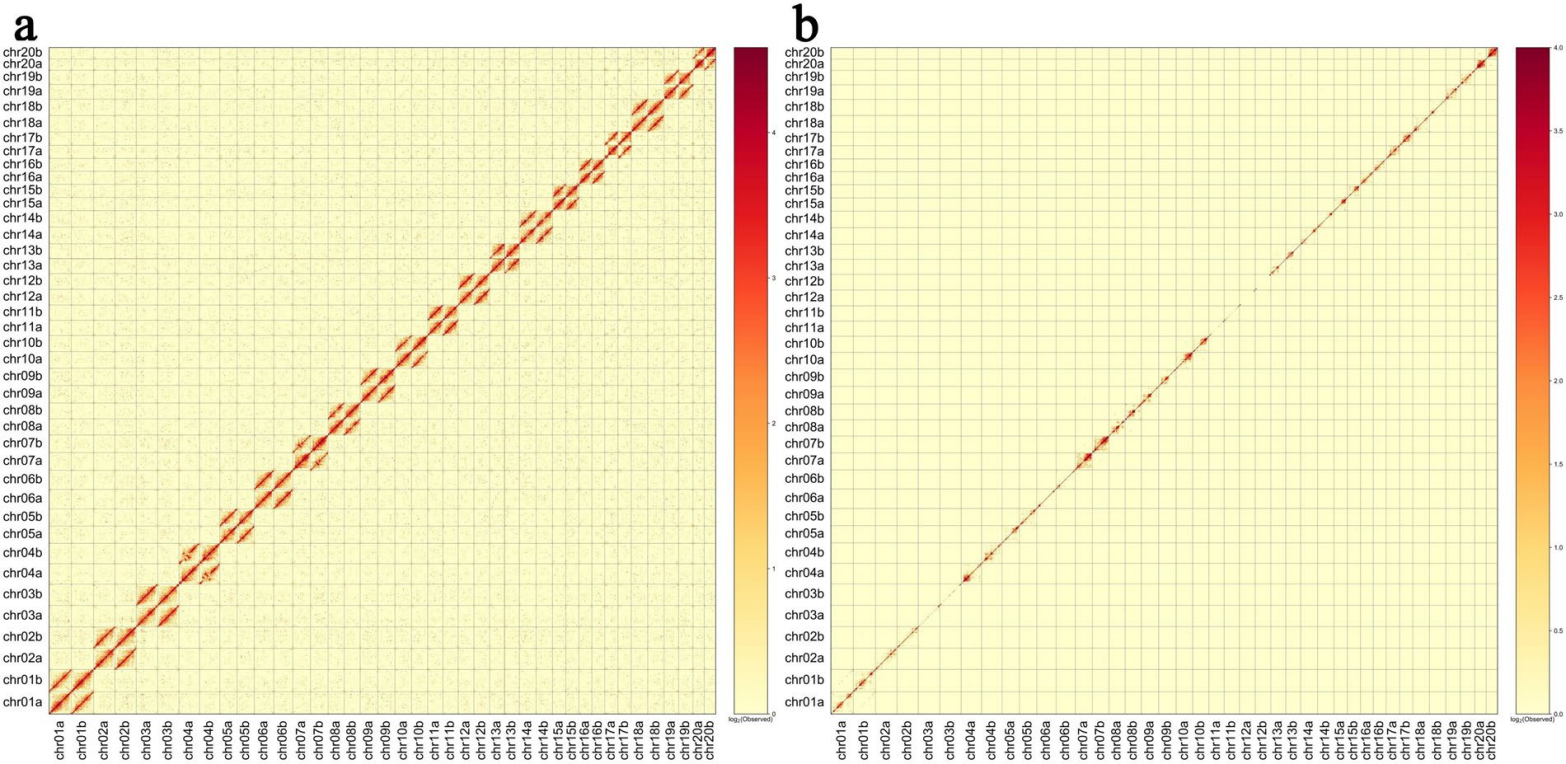
Hi-C interaction heatmaps for haplotypes A and B, depicting interactions for (**a**) reads with mapping quality of 0 or higher (including duplicates) and (**b**) mapping quality of 1 or higher (excluding duplicates). The color scale represents interaction intensity, with yellow signifying weak and red indicating strong interactions.

Hi-C reads were aligned to the genome assembly using default parameters. The pre command in Juicebox (pre -n -q 0 or 1) was used to convert the raw files generated by Juicer into the hic format, and the hic files were then visualized using Juicebox. The Hi-C interaction heatmaps generated by Juicer show high resolution for each chromosome (Fig. 5a), with no obvious noise observed outside the diagonals. Additionally, no anomalies were observed between each pair of homologous chromosomes (Fig. 5b), indicating no obvious switch errors.

In this study, the assembled genome has few gaps, high integrity of telomere sequences, high mapping rates, high completeness, no redundancy, and high-resolution Hi-C interaction heatmaps, all of which indicate that the quality of the genome assembly is close to that of a telomere-to-telomere (T2T) genome.

### Evaluation of the gene annotation

The quality of gene annotation was evaluated using BUSCO (Benchmarking Universal Single-Copy Orthologs) (v5.3.2)^68^, and the results are presented in Supplementary Table 7. The evaluation revealed that the complete core gene coverage was 98.8%, comprising 2.0% single-copy genes and 96.8% duplicated genes. The annotation was further characterized by a minimal presence of fragmented genes (0.3%) and missing genes (0.9%), collectively signifying a high-quality annotation.

## Supplementary information

Supplementary Information

## Data Availability

All commands and pipelines used were performed according to the manuals or protocols of the tools used in this study. The software and tools used are publicly accessible, with the version and parameters specified in the Methods section. If no detailed parameters are mentioned, the default parameters were used in this study. No custom code was written or used in this study.
