# Supplementary material for: A nearly telomere-to-telomere diploid genome assembly of Firmiana kwangsiensis, a threatened species in China
Source: Sci Data. 2024 Dec 18;11:1394. doi: 10.1038/s41597-024-04250-8 (PMC11655631; doi:10.1038/s41597-024-04250-8)
Supplement: Supplementary file 1 — Supplementary Information [file 41597_2024_4250_MOESM1_ESM.pdf]

Supplementary Figure and Tables

Table of Contents

Supplementary Figure 1. 3
Supplementary Table 1. 4
Supplementary Table 2. 4
Supplementary Table 3. 4
Supplementary Table 4. 4
Supplementary Table 5. 5
Supplementary Table 6. 6
Supplementary Table 7. 6

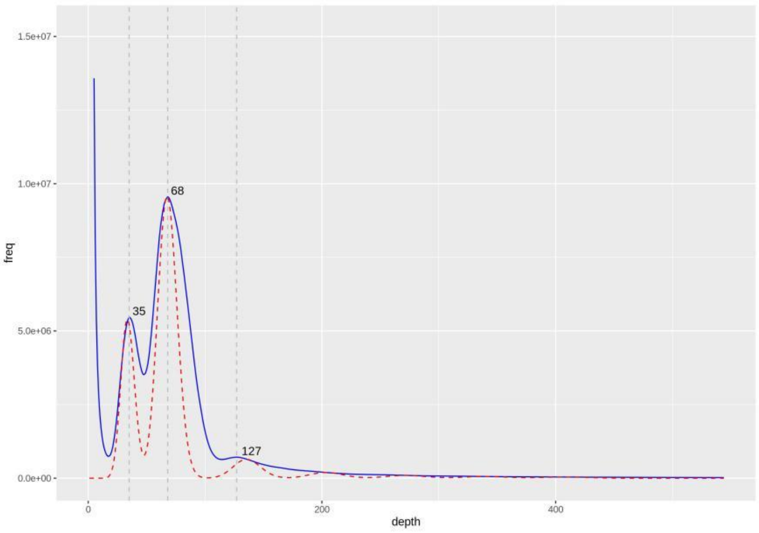

Supplementary Figure 1. The depth-distribution of 19-mers.

Supplementary Table 1. WGS-HiFi sequencing statistics.

**Total bases (bp)**	**GC content**	**A (bp)**		**T (bp)**		**G (bp)**		**C (bp)**		
83,637,081,877	37.41(%)	26,133,549,891 (31.25 %)		26,219,012,090 (31.35 %)		15,546,212,226 (18.59 %)		15,738,307,670 (18.82%)		
**Total reads**	**Max. (bp)**	**Mean (bp)**	**N10 (bp)**	**N50 (bp)**	**N90 (bp)**	**Min. (bp)**	**Median (bp)**	**L10**	**L50**	**L90**
4,483,876	55,951	18,652	24,854	18,541	15,092	506	17,842	308,620	1,903,166	3,905,712

Supplementary Table 2. ONT sequencing statistics.

**Total bases (bp)**	**GC content**	**A (bp)**		**T (bp)**		**G (bp)**		**C (bp)**		
46,941,501,005	37.96 (%)	14,466,215,202 (30.82 %)		14,657,084,477 (31.22 %)		9,206,630,656 (19.61 %)		8,611,570,670 (18.35%)		
**Total reads**	**Max. (bp)**	**Mean (bp)**	**N10 (bp)**	**N50 (bp)**	**N90 (bp)**	**Min. (bp)**	**Median (bp)**	**L10**	**L50**	**L90**
1,618,560	522,725	29,002	135,940	64,517	15,632	16	13,233	27,919	239,150	757,838

Supplementary Table 3. Hi-C sequencing statistics.

**Reads (M)**	**Bases (G)**	**Q20 (G)**	**Q30 (G)**	**Average length (bp)**
832.119	124.818	122.627 (98.2 %)	118.851 (95.2 %)	150

Supplementary Table 4. Iso-Seq data statistics.

**Total bases (bp)**	**GC content**	**A (bp)**		**T (bp)**		**G (bp)**		**C (bp)**		
18,726,456,249	42.22 (%)	5,320,606,619 (28.41 %)		5,499,786,293 (29.37 %)		3,940,290,752 (21.04 %)		3,965,772,585 (21.18%)		
**Total reads**	**Max. (bp)**	**Mean (bp)**	**N10 (bp)**	**N50 (bp)**	**N90 (bp)**	**Min. (bp)**	**Median (bp)**	**L10**	**L50**	**L90**
16,272,327	111,233	1,150	2,919	1,327	685	87	957	501,408	4,581,631	12,419,299

Supplementary Table 5. Summary of the basic features of the estimated genome based on kmer species (a) and kmer individuals (b).

K-mer	Raw peak	Now node	Low kmer	Now kmer	Coverage	Genome size (Mb)	Heterozygous ratio (%)		Duplication ratio (%)		Error_rate (%)
							a	b	a	b	
19	70	513,743,688	2,301,203,854	77,051,011,277	77.8153	1,043.92	1.075989342	0.236043132	15.1382	62.38115	0.170587465

Supplementary Table 6. Assessment of genome coverage rate.

Data set	Reads mapped	Bases mapped	≥ 1×	≥ 5×	≥10×	≥ 20×
HiFi	98.89 %	98.92 %	100.00 %	99.96 %	99.92 %	98.46 %
Iso-Seq	98.54 %	99.20 %	10.26 %	5.97 %	4.53 %	3.43 %

Supplementary Table 7. Assessment of BUSCO.

Category	Genome		Proteome	
	Number	Ratio	Number	Ratio
Complete BUSCOs	1,595	98.8 %	1595	98.8 %
Complete and single-copy BUSCOs	8	0.5 %	32	2.0 %
Complete and duplicated BUSCOs	1,587	98.3 %	1563	96.8 %
Fragmented BUSCOs	8	0.5 %	5	0.3 %
Missing BUSCOs	11	0.7 %	14	0.9 %
Total BUSCO groups searched	1,614		1614	
